# The complete mitochondrial genome of the lowland paca (*Cuniculus paca*) and its phylogenetic relationship with other New World hystricognath rodents

**DOI:** 10.1080/23802359.2023.2275830

**Published:** 2023-11-14

**Authors:** Juan Li, Pedro Mayor, Meddly L. Santolalla Robles, Alex D. Greenwood

**Affiliations:** a Department of Wildlife Diseases, Leibniz Institute for Zoo and Wildlife Research (IZW), Berlin, Germany; b Department of Biologie, Chemie, and Pharmazie, Freie Universität Berlin, Berlin, Germany; c Departament de Sanitat i Anatomia Animals, Universitat Autònoma de Barcelona, Bellaterra, Spain; d ComFauna, Comunidad de Manejo de Fauna Silvestre en la Amazonía y en Latinoamérica, Iquitos, Peru; e School of Public Health and Administration, Emerge, Emerging Diseases and Climate Change Research Unit, Universidad Peruana Cayetano Heredia, Lima, Peru; f School of Veterinary Medicine, Freie Universität Berlin, Berlin, Germany

**Keywords:** Mitogenome, hystricognath, *Cuniculus*, phylogenetics

## Abstract

The lowland paca (*Cuniculus paca*) is a nocturnal, widespread, and solitary large-sized rodent in the family Cuniculidae, and one of the most frequently hunted mammals in the Neotropical forests of Latin America. We assembled the first complete mitochondrial genome of lowland paca using three closely related hystricognath species as reference sequences. The mitochondrial genome is 16,770 basepairs (bp) in length, with similar characteristics of vertebrate mitochondrial genomes. We performed phylogenetic analyses using 26 mitochondrial genome of hystricognath species based on thirteen protein-coding genes. The result confirms the taxonomical placement among the New World hystricognath rodents with high support. The placement is consistent with previous phylogenetic studies based on individual mitochondrial and nuclear genes. The current study improves the phylogenic resolution of hystricognath rodents.

## 1. Introduction

**Figure 1.**
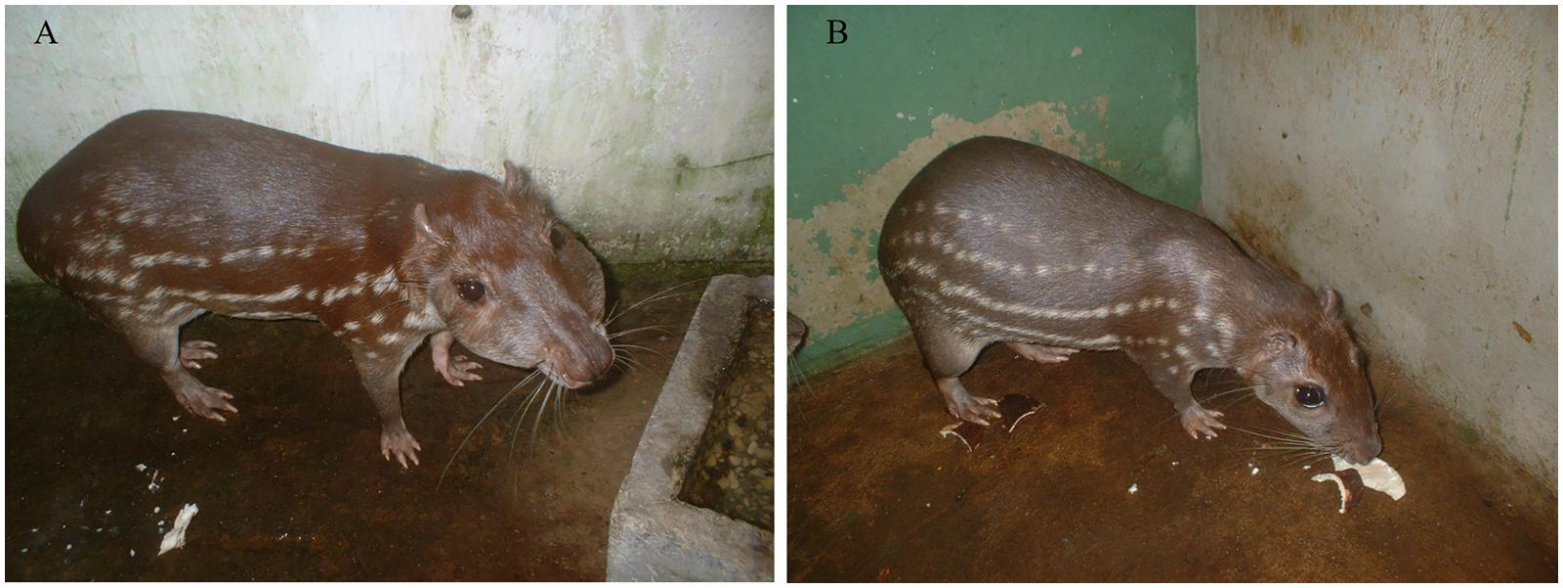
Images of male (A) and female (B) *Cuniculus paca* in captivity. The pictures were offered from the coauthor Pedro Mayor in this study.

The lowland paca (*Cuniculus paca*, Linnaeus, 1766) is the living species of *Cuniculus*, the only genus in the family Cuniculidae. The pacas were previously placed among the *Agouti* species in the family Dasyproctidae, genus Dasyprocta, subfamily Agoutinae, but has been defined as an independent family due to morphological differences (Grzimek 2003). Lowland pacas (Figure 1) are nocturnal, herbivorous and solitary large-sized (6–12 kg) rodents with a relatively low fecundity for rodents (Mayor et al. 2013, 2017). The species inhabit a variety of forest types in tropical areas within Central and South America from eastern and southern Mexico to northeast Argentina (Patton et al. 2015). Introductions into Cuba and the Lesser Antilles have occurred recently (Patton et al. 2015). Although this species is currently the most hunted Neotropical rodent species (El Bizri, Morcatty, Ferreira, et al. 2020; El Bizri, Morcatty, Valsecchi, et al. 2020), wild populations are stable and are listed as Least Concern by The IUCN Red List of Threatened Species in 2016 (Emmons 2016).

## 2. Materials and methods

A volume of ∼100 µl blood sample was collected by local hunters in the Yavarí-Mirin River basin (04°19′53″ S, 71°57′33″ W) in the Peruvian Amazon and was spotted on Whatman FTA card for storage. The specimen and DNA isolate were deposited in the frozen sample archive of Leibniz Institute for Zoo and Wildlife Research (IZW, https://www.izw-berlin.de/en/home.html; contact: Alex D. Greenwood, greenwood@izw-berlin.de) under the voucher number 151310178. We sliced the FTA card, which was diluted using AVL buffer in QIAamp Viral RNA Mini Kit (Qiagen, Hilden, Germany). After 10 min of vortex at 2000 rpm, genomic DNA was isolated using DNeasy Blood & Tissue Kit (Qiagen, Hilden, Germany) following the manufacturer’s protocol. Illumina DNA sequencing library following Meyer and Kircher (2010) was built with an average insert size of 300 bp, and was subsequently sequenced on the Illumina MiSeq platform using MiSeq Reagent Kit v2 (PE, 2 × 150 cycles).

We removed adapter sequences with Cutadapt v.1.15 (Martin 2011) and trimmed low quality reads with Trimmomatic v.0.38 (Bolger et al. 2014). Complete circular mitochondrial genome was assembled with MitoFinder (Allio et al. 2020) by using three complete mitogenomes of hystricognath species as reference sequences: *Cavia aperea* (NC_046949), *Cavia porcellus* (NC_000884), and *Coendou insidiosus* (NC_021387). All reads were then mapped backed to the *de novo* assembled mitogenome sequence using BWA v. 0.7.17 (Li and Durbin 2009). We called consensus sequence with default parameters using bcftools v.1.7 (Danecek and McCarthy 2017). The resultant mitochondrial genome was annotated using MitoFinder (Allio et al. 2020) after reverse complementing.

A phylogenetic analysis was performed using 13 PCGs of the lowland paca and 26 hystricognath rodents. Each gene from different species was aligned individually using MAFFT (-linsi) v7.310 (Katoh and Standley 2013). The multiple sequence alignments of the 13 genes were concatenated using catfasta2phyml (https://github.com/nylander/catfasta2phyml). A maximum-likelihood tree was estimated using IQ-TREE v.2.0.3 (Minh et al. 2020) with 1000 bootstrap replicates. We applied TVM + I + G as the substitution model for ND6 gene and GTR + I + G for other 12 genes. The substitution models were determined by PartitionFinder v.2.1.1 (Lanfear et al. 2016).

## 3. Results

**Figure 2.**
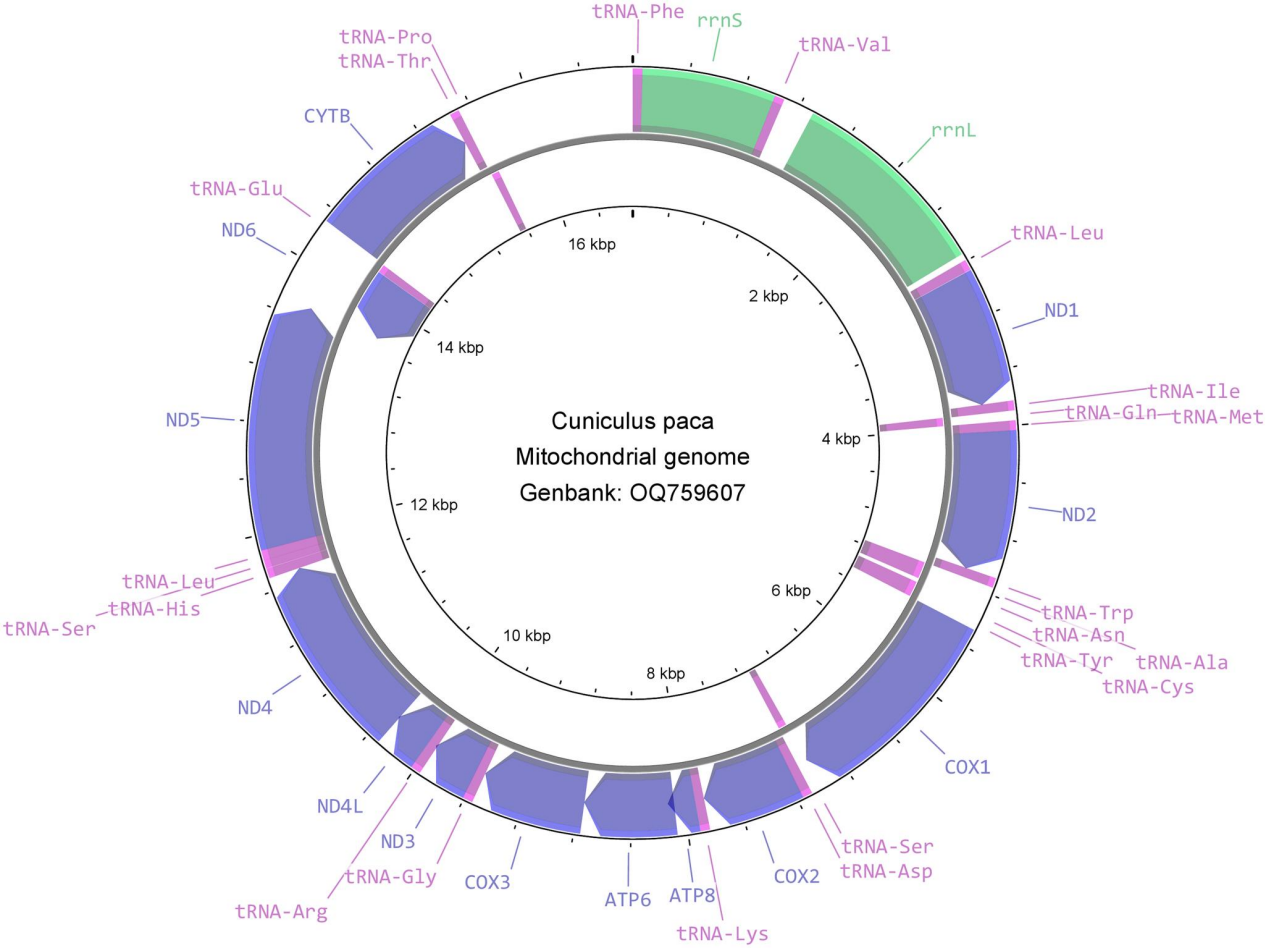
Clockwise view of the mitochondrial genome of the *Cuniculus paca* generated from CGView (Stothard and Wishart 2005).

**Figure 3.**
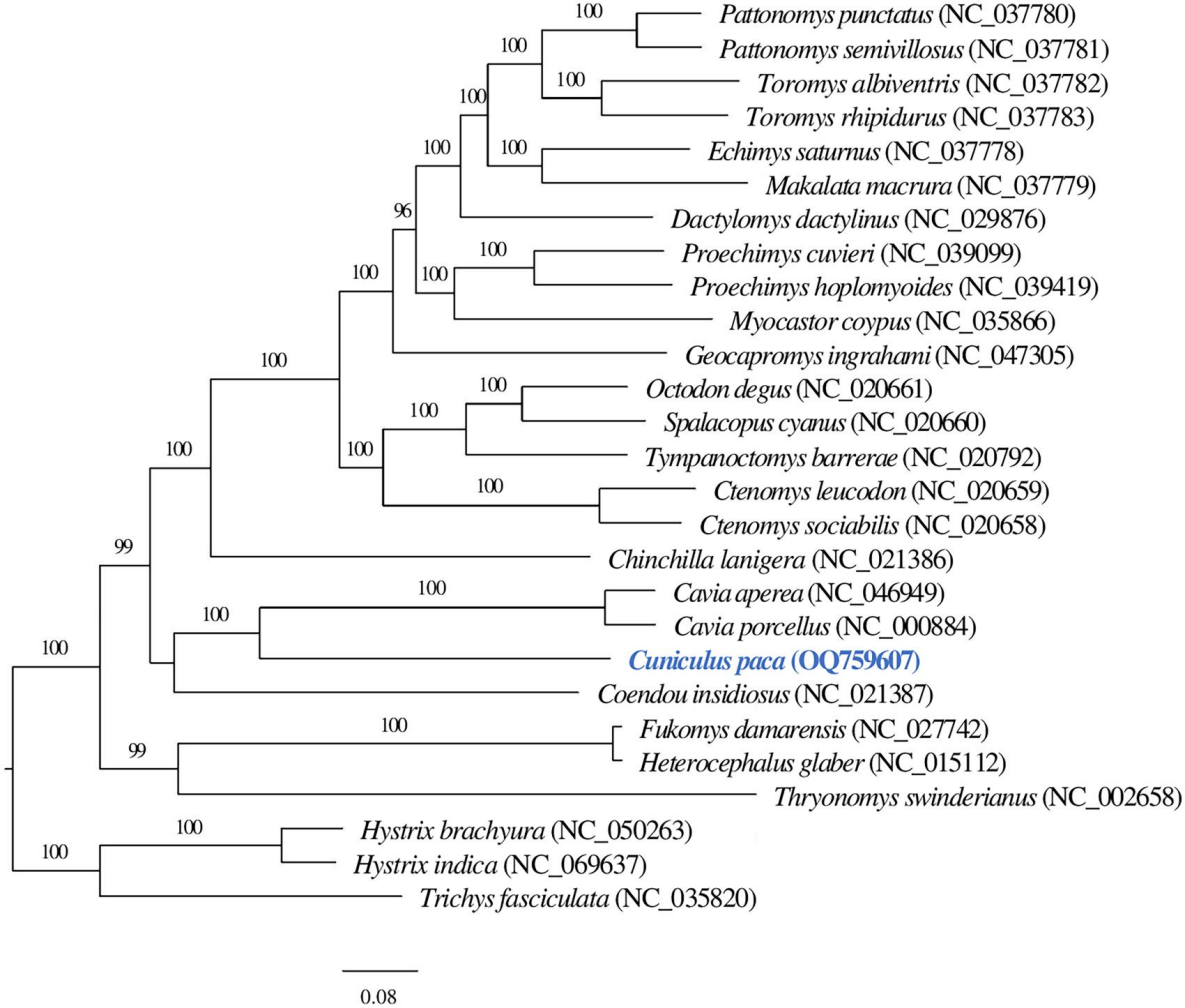
The phylogenetic relationship of lowland paca (*Cuniculus paca)* with 26 hystricognath rodent species are inferred from maximum-likelihood estimation based on 13 protein-coding genes with 1000 bootstrap replicates. Support values were given above each branch. The NCBI accession of mitogenome references used was displayed following species scientific names in parenthesis. The tip marked in blue depicts the mitochondrial genome of *Cuniculus paca* generated in this study.

In total, 45,552 merged reads out of 1,658,046 read pairs were mapped back to the assembled mitogenome sequence. The mitochondrial genome sequence has 100% total coverage with an average of ×370 base coverage, ranging from 28× to 1890× (Supplementary Figure 1). The complete mitochondrial genome of the lowland paca is 16,770 bp length with GC content of 40.81%, constituting a control region (position: 15,585–16,770) and a set of 37 genes: 13 protein-coding genes (PCGs), two ribosomal RNA (rRNA) genes, and 22 transfer RNA (tRNA) genes (Figure 2). The nucleotides of the mitogenome are composed of 32.98% adenine (A), 26.21% thymine (T), 13.33% guanine (G), and 27.47% cytosine (C). The majority of the PCGs initiate with the common vertebrate start codon ATG, except ND2 and ND3 with ATT, and ND5 and ND6 with ATC. Ten PCGs terminate with stop codons either TAG (ND1, ND2, COX1, ATP8, and ND3) or TAA (COX2, ATP6, ND4L, ND5, and ND6), while CYTB gene terminates with arginine coding codon AGA, COX3 with a single T and ND4 with TA. Phylogenetic analysis shows the lowland paca (*Cuniculus paca*) mitogenome clusters with the genus *Cavia* and is placed in the infraorder Hystricognathi within the hystricognath rodents (Figure 3).

## 4. Discussion and conclusions

Phylogenetic analysis confirms the taxonomical position of lowland paca (*Cuniculus paca*) mitogenome in the infraorder Hystricognathi within the hystricognath rodents, showing that *Cuniculus paca* is closely related to the genus *Cavia* (Figure 3). The results are consistent with previous analyses based on multiple mitochondrial and nuclear genes (Voloch et al. 2013). The current study *de novo* assembles a mitochondrial genome of lowland paca (*Cuniculus paca*) and improves the phylogenic resolution of hystricognath rodents.

## Supplementary Material

Supplemental Material

## Data Availability

The mitochondrial genome sequence supporting this study is publicly released at NCBI GenBank with the accession no. OQ759607. The associated sequencing data are deposited at NCBI Sequencing Reads Archive (SRA) under the BioProject PRJNA950048 (accession number SRR24006478 and BioSample number SAMN33969573).
