# Supplementary figures and images for: The complete mitochondrial genome of the lowland paca (Cuniculus paca) and its phylogenetic relationship with other New World hystricognath rodents
Source: Mitochondrial DNA B Resour. 2023 Nov 14;8(11):1220–3. doi: 10.1080/23802359.2023.2275830 (PMC10769523; doi:10.1080/23802359.2023.2275830)

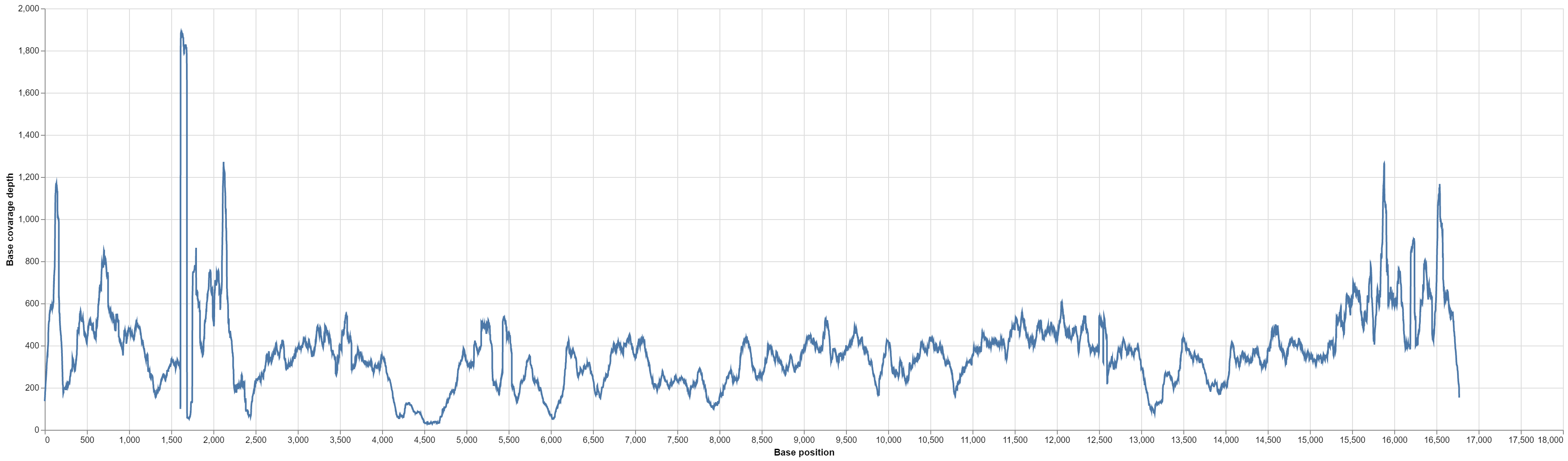

Supplement: Supplemental Material [file TMDN_A_2275830_SM7604.png]
